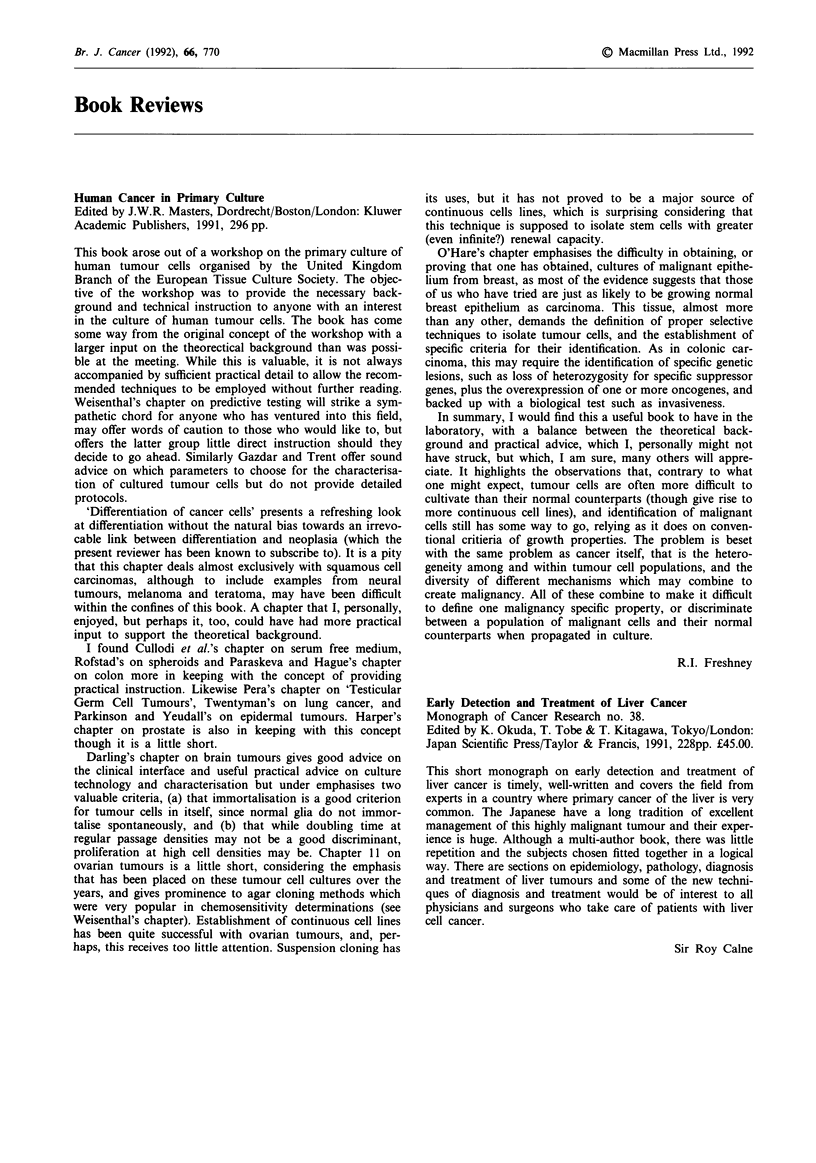# Human Cancer in Primary Culture

**Published:** 1992-10

**Authors:** R.I. Freshney


 © Macmillan Press Ltd., 1992

### Book Reviews

**Human Cancer in Primary Culture**
Edited by J.W.R. Masters, Dordrecht/Boston/London: Kluwer Academic Publishers, 1991, 296 pp.

This book arose out of a workshop on the primary culture of human tumour cells organised by the United Kingdom Branch of the European Tissue Culture Society. The objective of the workshop was to provide the necessary background and technical instruction to anyone with an interest in the culture of human tumour cells. The book has come some way from the original concept of the workshop with a larger input on the theorectical background than was possible at the meeting. While this is valuable, it is not always accompanied by sufficient practical detail to allow the recommended techniques to be employed without further reading. Weisenthal's chapter on predictive testing will strike a sympathetic chord for anyone who has ventured into this field, may offer words of caution to those who would like to, but offers the latter group little direct instruction should they decide to go ahead. Similarly Gazdar and Trent offer sound advice on which parameters to choose for the characterisation of cultured tumour cells but do not provide detailed protocols.

'Differentiation of cancer cells' presents a refreshing look at differentiation without the natural bias towards an irrevocable link between differentiation and neoplasia (which the present reviewer has been known to subscribe to). It is a pity that this chapter deals almost exclusively with squamous cell carcinomas, although to include examples from neural tumours, melanoma and teratoma, may have been difficult within the confines of this book. A chapter that I, personally, enjoyed, but perhaps it, too, could have had more practical input to support the theoretical background.

I found Cullodi *et al.*'s chapter on serum free medium, Rofstad's on spheroids and Paraskeva and Hague's chapter on colon more in keeping with the concept of providing practical instruction. Likewise Pera's chapter on 'Testicular Germ Cell Tumours', Twentyman's on lung cancer, and Parkinson and Yeudall's on epidermal tumours. Harper's chapter on prostate is also in keeping with this concept though it is a little short.

Darling's chapter on brain tumours gives good advice on the clinical interface and useful practical advice on culture technology and characterisation but under emphasises two valuable criteria, (a) that immortalisation is a good criterion for tumour cells in itself, since normal glia do not immortalise spontaneously, and (b) that while doubling time at regular passage densities may not be a good discriminant, proliferation at high cell densities may be. Chapter 11 on ovarian tumours is a little short, considering the emphasis that has been placed on these tumour cell cultures over the years, and gives prominence to agar cloning methods which were very popular in chemosensitivity determinations (see Weisenthal's chapter). Establishment of continuous cell lines has been quite successful with ovarian tumours, and, perhaps, this receives too little attention. Suspension cloning has its uses, but it has not proved to be a major source of continuous cells lines, which is surprising considering that this technique is supposed to isolate stem cells with greater (even infinite?) renewal capacity.

O'Hare's chapter emphasises the difficulty in obtaining, or proving that one has obtained, cultures of malignant epithelium from breast, as most of the evidence suggests that those of us who have tried are just as likely to be growing normal breast epithelium as carcinoma. This tissue, almost more than any other, demands the definition of proper selective techniques to isolate tumour cells, and the establishment of specific criteria for their identification. As in colonic carcinoma, this may require the identification of specific genetic lesions, such as loss of heterozygosity for specific suppressor genes, plus the overexpression of one or more oncogenes, and backed up with a biological test such as invasiveness.

In summary, I would find this a useful book to have in the laboratory, with a balance between the theoretical background and practical advice, which I, personally might not have struck, but which, I am sure, many others will appreciate. It highlights the observations that, contrary to what one might expect, tumour cells are often more difficult to cultivate than their normal counterparts (though give rise to more continuous cell lines), and identification of malignant cells still has some way to go, relying as it does on conventional critieria of growth properties. The problem is beset with the same problem as cancer itself, that is the heterogeneity among and within tumour cell populations, and the diversity of different mechanisms which may combine to create malignancy. All of these combine to make it difficult to define one malignancy specific property, or discriminate between a population of malignant cells and their normal counterparts when propagated in culture.

R.I. Freshney